# Calibration Method for Particulate Matter Low-Cost Sensors Used in Ambient Air Quality Monitoring and Research

**DOI:** 10.3390/s21123960

**Published:** 2021-06-08

**Authors:** Janani Venkatraman Jagatha, André Klausnitzer, Miriam Chacón-Mateos, Bernd Laquai, Evert Nieuwkoop, Peter van der Mark, Ulrich Vogt, Christoph Schneider

**Affiliations:** 1 Geography Department, Humboldt-Universität zu Berlin, Unter den Linden 6, D-10099 Berlin, Germany; andre.klausnitzer.1@geo.hu-berlin.de (A.K.); christoph.schneider@geo.hu-berlin.de (C.S.); 2 Department of Flue Gas Cleaning and Air Quality Control, Institute of Combustion and Power Plant Technology (IFK), University of Stuttgart, Pfaffenwaldring 23, 70569 Stuttgart, Germany; miriam.chacon-mateos@ifk.uni-stuttgart.de (M.C.-M.); bernd_laquai@i-tip.de (B.L.); ulrich.vogt@ifk.uni-stuttgart.de (U.V.); 3 Netherlands Organisation for Applied Scientific Research, Anna van Buerenplein 1, 2595 DA The Hague, The Netherlands; evert.nieuwkoop@tno.nl (E.N.); peter.vandermark@tno.nl (P.v.d.M.)

**Keywords:** air pollution, low-cost sensors, particulate matter, quantile mapping, mobile measurements

## Abstract

Over the last decade, manufacturers have come forth with cost-effective sensors for measuring ambient and indoor particulate matter concentration. What these sensors make up for in cost efficiency, they lack in reliability of the measured data due to their sensitivities to temperature and relative humidity. These weaknesses are especially evident when it comes to portable or mobile measurement setups. In recent years many studies have been conducted to assess the possibilities and limitations of these sensors, however mostly restricted to stationary measurements. This study reviews the published literature until 2020 on cost-effective sensors, summarizes the recommendations of experts in the field based on their experiences, and outlines the quantile-mapping methodology to calibrate low-cost sensors in mobile applications. Compared to the commonly used linear regression method, quantile mapping retains the spatial characteristics of the measurements, although a common correction factor cannot be determined. We conclude that quantile mapping can be a useful calibration methodology for mobile measurements given a well-elaborated measurement plan assures providing the necessary data.

## 1. Introduction

Epidemiological studies reveal that there is concrete evidence of the connection between poor air quality due to particulate matter (PM) pollution and health [1,2,3,4,5]. The United Nations Sustainable Development Goal 11 includes clean air as a basic human requirement for health and wellbeing and aims to reduce the per capita environmental impact, especially in urban surroundings [6]. The World Health Organization (WHO) classifies outdoor air pollution as a leading environmental cause of death due to cancer [7]. Air quality (AQ) awareness has also been rapidly increasing among citizens over the last decades [8]. Air quality monitoring stations (AQMS) run by institutions or governmental agencies are usually point-based and location-specific. The spatial coverage of such measurements is insufficient owing to the costs [9,10]. This fact, coupled with the advancements in micro-sensing technology, contributes to a paradigm shift from conventional air quality monitoring networks to rapid growth in air quality monitoring systems (AQMS) set up either by private initiatives or public institutions using low-cost sensors (LCS) which complement existing air quality monitoring networks [11,12]. Purple-Air [13] LCS company for instance works together with the Environmental Protection Agency of the United States of America (EPA) in its projects [14]. In Germany, Breeze Technologies [15] works on projects together with different state governments within their projects.

The EPA recognises the LCS for its versatility and has funded projects to assess the quality of the LCS and its applications [16]. The draft roadmap for the next generation of air quality monitoring from the EPA includes LCS [17]. The European Union’s science hub, the European Commission (EC) followed suit and launched its projects to determine the possibilities and limitations of LCS [18,19,20]. The Joint Research Centre (JRC) of the EC has taken it upon itself to test LCS and classify them according to their probable applications [21,22,23]. The JRC welcomes the frequent use of LCS by citizens, researchers, and institutions despite their drawbacks. Sense-Box project [24], supported by the Federal Ministry of Education and Research (BMBF), Germany, and the AirSensEUR project [25] of the JRC, indicate the recognition of the LCS by government organisations. The European Standardisation Organisation is working on a protocol [26,27] to evaluate LCS based on common criteria [28].

Over the last decade, many manufacturers have come forth with LCS for PM (PM-LCS) for measuring the ambient and indoor air quality. The costs range from EUR 15 to EUR 500 (see Section 2). PM-LCS are available as a unit produced by Original Equipment Manufacturers (OEM) and as a Sensor Box containing one or an ensemble of sensors produced by the OEMs. In addition, there are ‘do-it-yourself’ sensors available for LCS enthusiasts to custom design their sensor boxes. What these sensors make up for in cost, they lack in the reliability of the measured data due to their straightforward technological concepts, their sensitivities to temperature and relative humidity, and due to inappropriate usage when the sensors are deployed for studies outside of the manufacture specifications, for example, using an indoor sensor for outdoor measurements. In recent years a series of studies have been conducted to assess the possibilities and limitations of these sensors [29,30,31,32].

Moreover, the sensor market is extremely volatile, with new manufacturers entering the market and new sensors or upgrades of existing sensors hitting the market. This further complicates studies employing LCS as some of the available reports on specific sensors are becoming obsolete and new studies have to be carried out to assess the performance of newly released ones.

This study aims to review the calibration of PM-LCS as PM is one of the major air pollutants relevant in ambient air monitoring. It builds on a literature review and a wide survey among experts in the field in Germany amended by interviews in other countries of the European Union. Experts in the field were consulted for their experiences and recommendations. We further report and discuss own experiments for the calibration of mobile measurements with self-made PM-LCS measurement units. The sensors considered for this study are the ones that are currently being mostly used by citizen science initiatives and the scientific communities in Germany, but elsewhere as well and are defacto-standard in many comparison studies [11,12,33].

## 2. State of Art in PM-LCS Correction Procedures

The LCS technology and its usage among individual laymen, for example, within citizen science initiatives and researchers, are rapidly increasing. Since this is a relatively new technology with a highly volatile market, a lot of information is still available as grey literature and technical reports only [23]. The state of the art analysis of LCS in this chapter is provided in the form of a short overview of LCS technologies, a literature review, and from interviews with experienced and amateur researchers in the LCS area.

### 2.1. Principle Measurement Techniques for PM

Several methods, such as gravimetric sampling, photometry, and ß-radiation attenuation, are used to determine the concentration of PM in the air [34,35]. However, photometry using laser scattering is commonly used in most LCS due to its shorter response time and lower power requirement.

In reference-grade PM instruments, as explained in Baumbach (1996) [34], a beam of light is penetrated through an airflow channel (flue gas), and the extinction (logarithm of the ratio of emitted light intensity to the attenuated light intensity) is measured. According to the Lambert-Beer law [36], extinction is directly proportional to the concentration of interest in the flue gas. However, a light beam sent through a gas volume laden with particles is not only attenuated but also scattered. This phenomenon of scattering of light by particles is used in ambient air measurements. In light-scattered photometry, a beam of light is sent by the emitter to a flicker mirror which alternatively generates a measuring beam and a reference beam. The particles from the air sucked into the system scatters the measuring beam which is detected by the photocell. The amount of scattered light is measured in comparison to the reference beam which is indicative of the concentration of the particles in the air.

Continuously measuring PM instruments, such as the Grimm 1.108/1.109 (henceforth referred to as Grimm), for example, use the Mie-Scattering principle, in addition, to enable particle sizing [37]. This is carried out in Grimm by using a semiconductor laser as a light source and a measuring cell wherein the scattered light is led directly and via a mirror onto a detector. The scattering light pulse of every single particle is counted along with the intensity of the scattered light signal giving rise to both particle counts and particle sizing, respectively. An accurate sampling volume of 1.2 L/min is used by Grimm to enable precise and reproducible particle counting and particle sizing [37].

In PM-LCS such as the OPC series (OPC-N2, OPC-N3, and OPC-R1) from Alphasense Ltd. [38]., Nova SDS011 (SDS011) [39], SPS30 from Sensiron GmbH [40] operate using the laser scattering principle, similar to that of Grimm. Alphasense replaced the powerful pump and measurement cell in Grimm with a mircrofan leading to a virtual sensing zone [38]. It measures the particles and creates a size-distribution. The mass concentration is then obtained using an algorithm using the number concentration, refractive index, particle density and a weighting-factor. Alphasense uses a particle density of 1.65 g/mL but provides an option to alter the particle size density for each bin according to the need of users and the refractive index of particles. SDS011 and SPS30 do not specify how a “measurement cell” is created within their sensors. SDS011 and SPS30 use algorithms to calculate the mass concentration for PM10 by using the particle number concentration measured, an assumed particle size distribution, and particle density. An in-depth discussion of the measurement principles of most LCS is provided in Alfano et al. (2020) [41].

The information available from the datasheets of the most popular PM LCS sensors are summarised in Table 1 and Table 2. Table 1 lists the manufacturer, model number, the dimensions of the sensor in mm, measurement principle used (laser scattering spectrometer, LSS, or photometer), measurement and detection ranges, time resolution (T.R.), and the approximate cost in Euro (€). Table 2 dives deeper into the information on the electrical and performance characteristics of the sensors mentioned in Table 1 and lists the nominal voltage (V) and maximum power consumption in Watts (W), operating temperature, and relative humidity range under condensing or non-condensing (n.c.) conditions, uncertainty, sensor life, availability of calibration, and the reaction time in seconds (s). Information on the repeatability and drift was not available for any of the sensors. “NA” is used to indicate when information was unavailable.

The SDS011 from Nova-Fitness Ltd., China, the PMS series from Plantower Technology, China, and the OPC series from Alphasense Ltd., UK, are some of the most popular choices of LCS. The SDS011 costs around EUR 30 without additional electronics to capture and store data. It is one of the most used sensors in citizen science projects such as the Sensor Community project in Europe [42]. The Plantower sensors are widely used in the USA in research [43] and in citizen science initiatives such as the CityOS project [44]. The OPC series, on the other hand, costs between EUR 300 and EUR 450 and is used more in research work. The extra costs compared to, for example, the SDS01 sensor is compensated by the ability of the OPC to provide a histogram of particle-sizes in 16 bins (OPC-N2) or 24 bins (OPC-N3 and OPC-R1) and PM1 in addition to the overall mass distributions of PM10 and PM2.5. The SPS30 (Sensiron AG, Zürich, Switzerland) is gaining popularity but the literature available on this sensor still is very limited.

The cost-effectiveness of LCS comes with its own disadvantages. The inherent limitations of the PM sensors, when compared to expensive reference devices, introduce variations in the measurements between the two devices. By construction, an expensive standard device such as a Grimm Aerosol Spectrometer has an advantage over a LCS due to the presence of a pump. Even though a ventilator is often present in LCS such as the Alphasense family and SDS011 (Nova Fitness) sensors, the power of such a ventilator is quite low as it produces a mere 300 mL/min or less sample flow rate compared to the 1.2 L/min sample flow rate of a Grimm 1.109/1.108. Conventional OPCs also have a narrow air inlet that leads to the centre of a measurement chamber wherein the air sample is illuminated with a laser source in a multiplex mode. This means that the laser intensity is modulated, enabling the ability of the instrument to measure a wide range of particle sizes [37]. The OPC-N2 has its patented system, wherein the expensive pump and narrow inlet are replaced with a micro-fan which sucks in the air into an open scattering chamber, wherein an elliptical mirror and a dual-element photodetector create a “virtual sensing zone” where the laser light illuminates, scatters and is detected. The smaller sized particles are calculated using a weighing to account for their underestimation in LCS [38].

LCS are generally not stand-alone instruments, which means that they need additional electronics for power supply, configuration, and data storage. However, the OPC-N2 is a stand-alone instrument with software included which runs on a Windows operating system [45] and uses an internal SD card to store data. However, the instrument by itself is not weather-proof nor does it come with temperature and RH sensors, a clock module, or a global navigation satellite system (GNSS) receiver. Therefore, the OPC-N2 has to be set up with additional microcomputers such as the Raspberry Pi [46] or Arduino [47] or with a custom-made printed circuit board (PCB). However, the latest version, OPC-N3, comes with built-in temperature and RH sensors.

### 2.2. Literature Review on PM-LCS Calibration Studies

Lukeville (2019) [8] has undertaken a comprehensive overview of the basic principles involved in the LCS measurement technology, ensuring their quality, reliability, and limitations. She also provides information on how the air quality is measured in Europe and how citizens can calibrate their sensors using existing official stations, and how they might provide the data to the authorities for further use.

Due to the widespread and increasing popularity of LCS among scientists and citizens, Aakash C. Rai et al. (2016) [21] and Aakash C. Rai and Prashant Kumar (2017) [22] provide an overview of available stand-alone sensors, their technology, and the costs involved. Updated and exhaustive information on not just stand-alone sensors but of sensor systems, including black-box sensors, was carried out by the JRC [23]. This study provides quantitative data on the performance of LCS against reference instruments. It is concluded that the coefficient of determination (R^2^) metric, used by most studies that evaluate a sensor’s capabilities, can be misleading on the quality of LCS. This is because the R^2^ is overly dependent on a range of different reference measurement specifications on the duration of the test, and the season and location of the test, making the changes in R^2^ not completely dependent on the LCS data quality or the calibration methods alone. Due to these shortcomings, Karagulian et al. (2019) [23] state that the standardization of a protocol for the evaluation of LCS has a high priority at an international level.

Kuula et al. (2020) [33] investigate the particle-size selectivity and its role in the analysis of sources of errors in LCS. They report that six sensors, namely the Plantower PMS5003, Nova SDS011, Sensiron SPS30, Sharp GP2Y1010AU0F, Shinyei PPD42NS, and Omron B5W-LD0101, are compared against a Grimm 1.108 (2020) with a vibrating orifice aerosol generator 3450 (VOAG, TSI Inc., Shoreview, MN, USA). The results show that none of the sensors adhere to the detection ranges claimed by the manufactures. In comparison with the Grimm 1.108, the sensors could achieve comparable data in one or two size bins only, which is insufficient for a sensor to be able to provide reliable mass concentration data.

Sousan et al. (2016) [48] compared the Alphasense OPC-N2 to the Grimm PAS 1.108 with the SMPS C5.402 (Grimm Aerosol GmbH, Ainring, Germany) and APS 3321 (TSI Inc., Shoreview, MN, USA) as reference instruments. Salt, welding fume, and Arizona street dust were used as input aerosols in an experimental setup, and the detection efficiency, response, and precision of both number concentration and mass concentration were assessed. For all the aerosols and PM metrics, the firmware-calculated mass concentrations had an R^2^ value of 0.97, whereas the number concentrations were found to be underestimated in the lower particle-size range (salt and welding fumes) and overestimated for coarse particles (Arizona street dust) when compared to reference instruments. The two OPCs, OPC-N2 and Grimm PAS 1.108 themselves were found to be consistent with each other.

Official air quality monitoring stations (AQMS) use accurate, but expensive devices. This makes it difficult to set up multiple stations to allow higher spatial resolution. LCS can come in handy in such situations by complementing AQMS. However, such setups only return reliable measurements when the LCS is well calibrated and extensive post-processing of the measured data is carried out. Di Antonio et al. (2018) [49] and Crilley et al. (2018) [50] provide such a correction methodology for LCS. Di Antonio et al. (2018) [49] for instance use the measured particle size distribution of the OPC-N2 sensor instead of the mass concentration to derive a correction based on relative humidity (RH) for individual particle sizes due to the hygroscopic properties of the dust particles. This is done by using Koehler’s theory [51,52], see also Section 3.3, which can significantly improve sensor performance and retain information on particle composition. The algorithm provided is also flexible to changes in particle chemical composition and particle chemical speciation.

However, not all sensors in the market have the ability to provide particle size distribution. Most of the LCS widely used in citizen science projects output only the standard mass concentration of PM of aerodynamic diameter 10 µm (PM10), 2.5 µm (PM2.5), and 1 µm (PM1). Sensiron’s SPS30 produce the mass concentration of PM of aerodynamic diameter 4 µm (PM4) in addition to the standard mass concentrations. When an LCS measures only the bulk PM it is difficult to implement a correction based on particle size. However, when the information from all bins is available, as is the case of the OPC series, it is possible to calculate the mass concentration using its own correction factors. Therefore, Crilley et al. (2018) [50] have outlined a simple correction factor as reported in the following paragraph, also based on the Koehler’s theory which is briefly described in Section 3.3. A number of 14 instruments were used in their study. All 14 instruments were co-located and then deployed. The instruments showed reasonable inter-unit precision and a reasonable agreement to reference optical-particle counters, TEOM-FDS, Grimm PAS 1.108, and TSI 3330, under low-to-normal RH. Under high ambient RH (>85%) a significant positive artefact was detected, which reiterates the necessity to correct the measured data for ambient RH. To correct for the ambient RH two aspects are to be noted here. First, the mass concentrations as computed by the OPC-N2 using factory-set algorithms are ignored. They are instead calculated from the particle size distribution (particle number concentration in different size bins) data of the OPC-N2 and the reference instruments using a uniform particle density of 1.65 g/mL (factory setting for OPC-N2). Second, applying this correction for RH < 85% tends to overcorrect the data. Crilley et al. (2018) [50] also note that “all low-cost PM sensors will likely require calibration factors to obtain the dry particle weight unless they actively dry the PM-containing air stream before it enters the device”. They also hint at the use of heated inlets to reduce the RH in the air stream. Samad et al. (2021) [53] investigated the use of a low-cost dryer for the OPC-N3, concluding that it can successfully reduce the negative effects of the relative humidity on the PM results. However, this alters the power requirements of the sensor with the consequence that either larger batteries are needed or only shorter operation times can be accomplished for off-grid operations.

Crilley et al. (2020) [54] checked the validity of the calibration method described in Crilley et al. (2018) [50] in four cities and on three different continents. They report that the elevated particle mass concentration found in LCS is due to the bulk aerosol hygroscopicity under different RH conditions. Crilley et al. (2020) [54] conclude that a factor based on Koehler’s theory (ĸ-factor) derived from in situ measurements, as they did, offers better calibration and improves the performance of the OPC-N2. Nevertheless, in conditions where in situ measurements are impractical, then Crilley et al. (2020) [54] suggest using a “literature-based ĸ-factor”.

Laquai and Saur (2017) [55] explain a calibration strategy for PM2.5 measured using the SDS011 sensor and using the Grimm 1.108 as a reference instrument. Based on an experimental setup, it is found that the PM10/PM2.5 ratio of the LCS gives an indication of the particle mass distribution. With this information, a range of ratios between the LCS sensor and Grimm for PM10 and PM2.5 is obtained in different particle spectra and a correction algorithm is deduced.

Datta et al. (2020) [56] describe a calibration method using a gain-offset model and linear regression for PM2.5 measurements. This study is an evaluation of a cluster of 32 LCS at one regulatory site. The evaluation was carried out using multiple linear regressions with co-located data.

Zusman et al. (2020) [43] evaluated the performance characteristics of two LCS, Plantower PMS A003 and Shinyei PPD42NS, in comparison to reference methods. They developed a regional calibration model for seven metropolitan areas in the United States of America. They observed occasional spikes of PM2.5 concentrations when the sensors warmed up, which led to excluding the first 8 h of data after each deployment.

A common trend observed in all the literature is that they all agree that the LCS can be a very important and useful commodity to complement existing AQMS, provided they follow proper measurement practices, are compensated for effects of varying RH, and apply data processing techniques. Nevertheless, the data processing in most of the LCS relies on setting up a mathematical model to fit the data of the LCS to a reference device. The regression model is usually the model of choice for LCS calibration [22,57]. However, these studies focus on point-based stationary measurements, integrated over a longer period of time (>=60 min). Mahajan and Kumar (2020) [58] observed that support vector regression (SVR) appeared to be a promising approach to calibrate LCS when compared to linear regression, artificial neural networks, and random forest regression. However, the scope of their study is also limited to stationary measurements. The validity of such models with respect to seasonal changes is not mentioned in these studies. Alfano et al. (2020) [41] provide the most recent and extensive review of LCS and their calibration. They emphasize the strong dependency of the performance accuracy of LCS on whether the device is calibrated or not in the operative environment. Therefore, using co-located calibrations to determine the accuracy of LCS on mobile platforms fails to account for the micro-scale changes in the spatial characteristics that further affect the accuracy of LCS.

### 2.3. Interviews on Usage and Calibration of LCS

As a part of a project on developing a communication strategy for citizen science projects and the general public on the usage of LCS for the German Environmental Agency (Umweltbundesamt, UBA) a series of 18 interviews were conducted between October 2019 and August 2020 with researchers working with LCS. The list of participants can be found in Appendix A. The following conclusions were drawn from the interviews:

- Expert interviews show a lack of uniformity in the testing of sensors. New guidelines are needed to make sensor testing procedures binding and comparable;
- When using sensors, it is important to be clear about what they are to be used for. If the aim is to increase the environmental awareness of citizens or to test the air quality (low pollution, high pollution) in a location, the quality of the data is sufficient. Currently, the raw data of the sensors are not suitable for quantitative measurements due to their poor reproducibility and stability characteristics;
- Many research groups have used the sensors without calibration. The number of calibrations required during a measurement campaign is still unclear. Most research groups carry out the calibrations in comparative measurements with standard measuring instruments at the beginning, when the measurement campaign is short, and additionally at the end in longer measurement campaigns;
- The data sheets provided by the manufacturers are partly insufficient. Therefore, calibrations by the user are essential. In addition, each sensor must be calibrated separately, since the characteristics of the sensors are individual even with sensors of the same type;
- A big issue is that LCS are operated outside their specifications. Almost all require a non-condensing environment. LCS are mostly sensors developed for indoor use. In many cases these sensors are used for outdoor measurements, thus failing to provide useable data;
- Single laboratory or co-location experiments are insufficient to determine the measured values and characteristics of the sensors. If the sensors are to be used for mobile measurements, stationary calibrations are insufficient. Furthermore, the age-related drift of the sensors must be taken into account. The service life of the sensors is usually less than specified by the manufacturer;
- A common platform for users of low-cost sensors for communication and exchange of information and ideas is indispensable. The circle of users of such low-cost sensors is constantly growing in private and commercial applications as well as in science without proper assurance of quality and information regarding visualization and interpretation of such measurements;
- Nevertheless, citizen scientists and the general public should be encouraged and guided to work with LCS and the data acquired through them.

## 3. The URBMOBI 3.0 System for Mobile PM Measurements

### 3.1. Device Configuration of the URBMOBI 3.0 System

**Figure 1:**
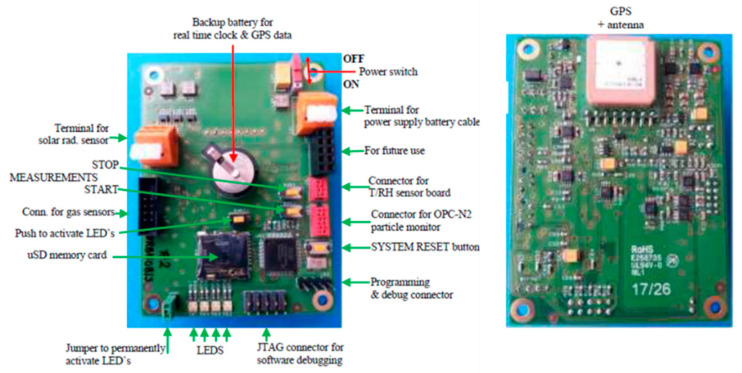
Printed circuit board (PCB), bottom-side (**left**) and top-side (**right**), of the URBMOBI 3.0 sensor-box. Photo: Evert Nieuwkoop.

In order to have a sensor box on a mobile platform, a sensor ensemble was designed and set up with a custom-made PCB called the URBMOBI 3.0, short for URban MOBIle instrument in its development phase 3, building on earlier versions 1.0 and 2.0 [59]. URBMOBI 3.0 is equipped with a PM [38], nitrogen dioxide (NO_2_) [60], nitrogen oxide (NO) [61], ozone (O_3_) [62], global radiation [63], and two temperature and RH [64] sensors (Figure 1).

**Figure 2:**
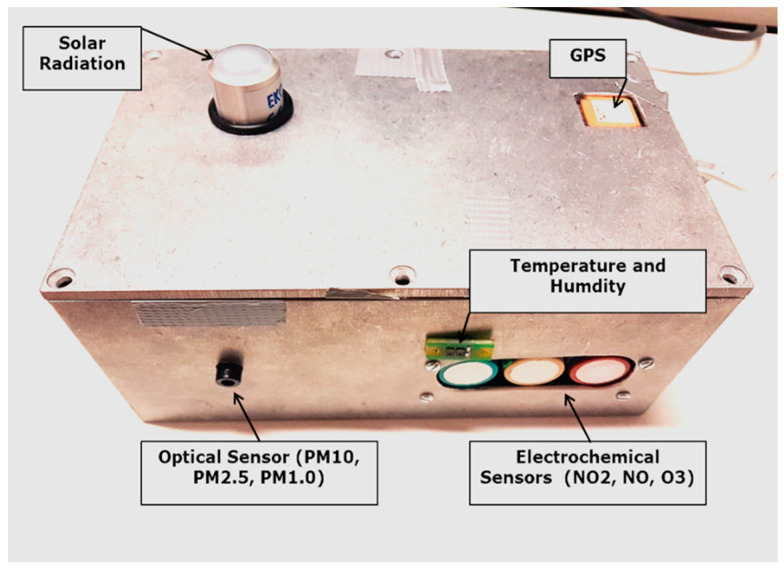
URBMOBI 3.0 device for mobile air quality and meteorology with sensors for fine dust (OPC-N2), gases (NO_2_, NO, and O_3_), air temperature, and humidity (SHT35), and global radiation (EKO ML-01); Photo: Janani Venkatraman Jagatha.

The URBMOBI 3.0 PCB is equipped with a GNSS receiver and an SD card to store data offline with a time resolution of 2 s. Figure 2 shows the setup of the URBMOBI sensor ensemble in its metal box. The instrument is not completely protected against rain and spray water and in such cases, the measurements should be stopped.

### 3.2. Calibration of the URBMOBI 3.0 System in a Stationary Setup

**Figure 3:**
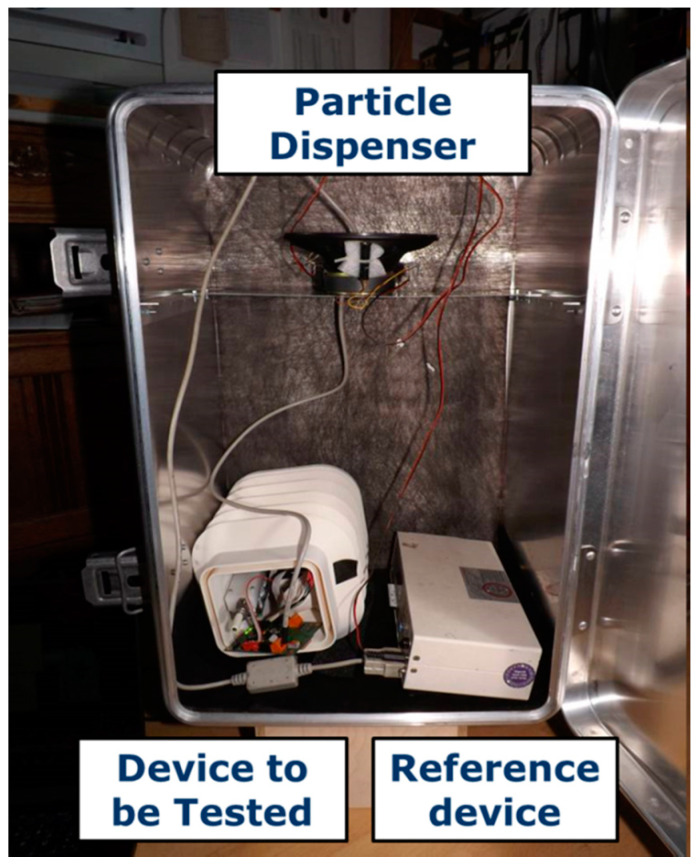
Self-built particle generator as measuring setup for comparison measurements between a reference instrument (Grimm 1.108, bottom right) and URBMOBI 3.0 (bottom/middle left); Photo: Bernd Laquai.

**Figure 4:**
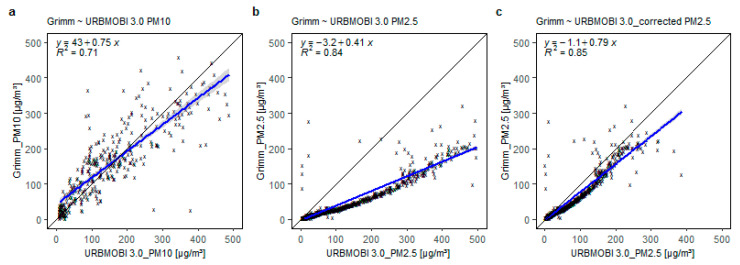
Results of the calibration tests recorded by the URBMOBI 3.0 (metal housing) and the reference instrument (Grimm 1.108) inside the particle generator with a time integral of 6 s; (**a**) PM10 scatter plot between Grimm 1.108 raw data and URBMOBI 3.0; (**b**) PM2.5 scatter plot between Grimm 1.108 and URBMOBI 3.0 raw data; (**c**) PM2.5 scatter plot between Grimm 1.108 raw data and URBMOBI 3.0 data corrected using the compensation function.

As a first step, the LCS is calibrated against a reference device, Grimm 1.108, in a stationary setup. The setup and procedure were followed according to Laquai et al. (2020) [65]. Figure 3 shows the test setup using a particle generator. The particle generator is a simple Zarges™ box acting as a particle chamber. The top portion of the box is provided with a loudspeaker and a smoke generator. Flour is used as a source of particles greater than 2.5 µm and is dispensed with the help of the loudspeaker. The smoke generator is used to dispense particles with less than 2.5 µm aerodynamic diameter. Both particle dispensers are connected and operated via external circuitry. The bottom part of the box is fitted with a Grimm 1108 device as the reference and space to place the LCS to be tested. The entire experiment is carried out under constant temperature (≈20 °C) and RH (≈50%) conditions. The results of the experiment are presented in Figure 4.

From the tests in the particle generator, it is observed that for particles above 2.5 µm aerodynamic diameter, the URBMOBI 3.0—OPC-N2 and the reference device are comparable. However, for smaller particles (<2.5 µm aerodynamic diameter) the URBMOBI 3.0—OPC-N2 tends to overestimate partial mass concentration when compared to the reference device. This means that a size-dependent compensation function is necessary to calibrate the OPC-N2 against the reference device, assuming the reference device to be more accurate in an absolute sense. The compensation function is generated by assuming a linear relationship between the URBMOBI 3.0—OPC-N2 (LCS) and the reference device:

(1) $$PM2.5_{Ref}=a*\;PM2.5_{LCS}+C,$$

where $PM2.5_{Ref}$ is the PM2.5 concentration of the reference device (Grimm 1.108), $PM2.5_{LCS}$ is the PM2.5 concentration of the URBMOBI 3.0—OPC-N2 (LCS), “*a*” denotes slope, and “*C*” the constant in the calibration equation. The assumption of a linear relationship is because constant temperature and RH conditions are maintained. The result of the compensation function is provided in Figure 4c. It is apparent that after applying this correction the OPC-N2 in the URBMOBI 3.0 ensemble is well suited for both PM10 and PM2.5 measurements under such dry and constant conditions.

**Figure 5:**
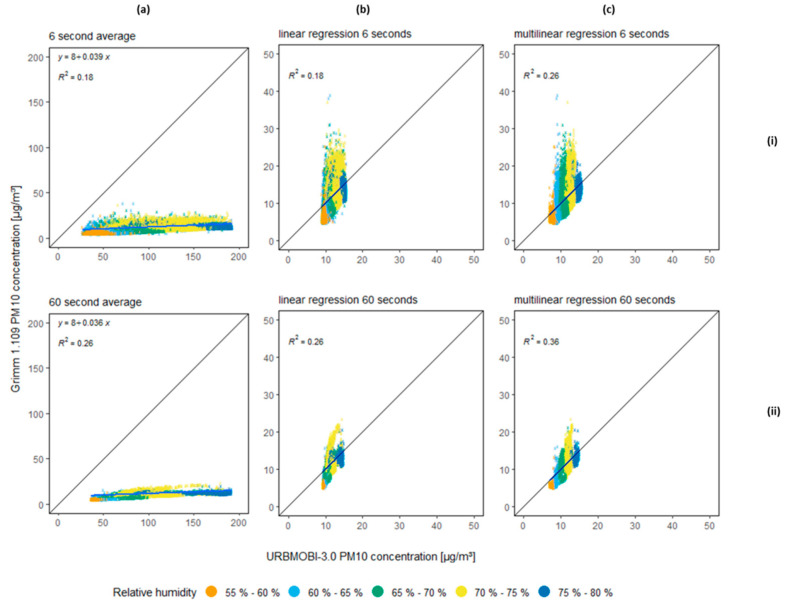
URBMOBI 3.0 (LCS) vs. Grimm 1.109 (reference device) PM10 comparison at Berlin-Adlershof; (**a**) scatter plot between the Grimm 1.109 raw data and URBMOBI 3.0 raw data with a time integral of 6 (**i**) and 60 (**ii**) seconds; (**b**) scatter plot between the Grimm 1. 109 raw data and URBMOBI 3.0 data corrected with linear regression with a time integral of 6 and 60 s (**c**) scatter plot between the Grimm 1.109 raw data and URBMOBI 3.0 data corrected with multi-linear regression, using temperature and RH as explaining variables, with a time integral of 6 and 60 s.

However, the goal for the URBMOBI 3.0 instrument is the implementation of an LCS to measure the ambient particulate matter concentration. Therefore, a second test with the LCS setup was carried out outdoors with a Grimm 1.109 as a reference device. The test was carried out in the Adlershof suburb of the Berlin metropolitan area between 2020–11–12 22:00:00 UTC and 2020–11–15 12:00:00 UTC at an altitude of 35 m, away from the road, on the building of the Geography Department of Humboldt-Universität zu Berlin. The air inlet of the reference device was at a height of 1.5 m and the air inlet of the LCS was at a height of 1.25 m above the floor of the roof. During the measurement period, the ambient temperature (T) ranged between 8.5 °C to 18 °C and the relative humidity (RH) between 55% to 80%. It can be seen in Figure 5a that the sensor clearly overestimates PM10 concentrations as compared to the data measured using the Grimm 1.109 device. The gain of the LCS increases as the RH increases (Figure 5). Furthermore, Figure 5a–ii and Figure 5c–i, both have the same coefficient of determination ($R^{2}$) value, even though Figure 5a–ii integrates the data over time (60 s) while Figure 5c–i keeps the 6-s interval but performs a multilinear regression using the temperature and relative humidity as explaining variables. Comparing Figure 5a–ii and Figure 5c–i, it is clear that a simple integration over longer periods is insufficient. It is important to consider the effects of the temperature and RH to explain and calibrate LCS.

(2) $$PM10_{Ref}=a*\;PM10_{LCS}+b*T+c*RH+C,$$

where *a*, *b*, and *c* are the slopes of *PM10_LCS_*, *T*, and *RH* respectively.

It is observed that PM10 also needs to be corrected under ambient conditions, as opposed to just PM2.5 as found in the laboratory experiment using constant air conditions and a particle generator. This means that the URBMOBI 3.0 instrument is in principle well suited for ambient measurements, provided a careful and well-designed cleaning and processing of the data is carried out. Single values are unreliable, especially when a high temporal resolution is used. Therefore, it is highly recommended to make temporal integrals over at least 60 s which is also evident from the comparison of Figure 5c–i and Figure 5c–ii.

### 3.3. Calibration of the URBMOBI 3.0 in a Mobile Setup

**Figure 6:**
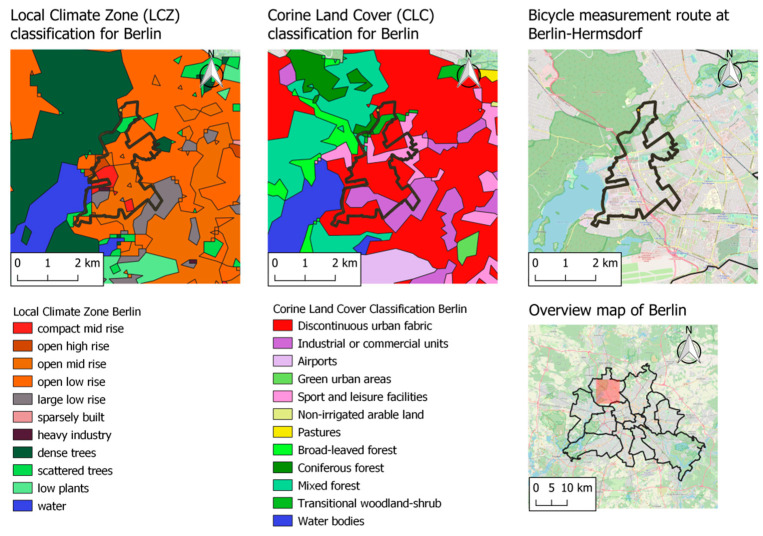
Measurement route at Berlin-Hermsdorf across different local climate zones and land use classes.

The URBMOBI 3.0 is envisioned to be deployed for mobile applications. In addition to hygroscopic effects, the LCS has to be proofed for sensitivities of the photodetector, angle of the laser, electronic defects, or vibrations causing a systematic error. To compensate and correct for the uncertainties arising from these factors and to evaluate the performance of the URBMOBI 3.0 in a mobile setup, a Grimm 1.109 instrument was carried additionally as a reference device, on a predetermined bicycle route. The measurements were carried out along an 18 km route, covering different local-climate zones (LCZ) [66] and land-use classes (LUC) as classified in the Corine land classification (CLC) [67] in the North-Western part of Berlin, Germany. Each measurement round took approximately 1.5 h to complete with the bicycle ridden at a mean speed of 15 km/h. Figure 6 shows the route across the various LCZ and LUC.

**Figure 7:**
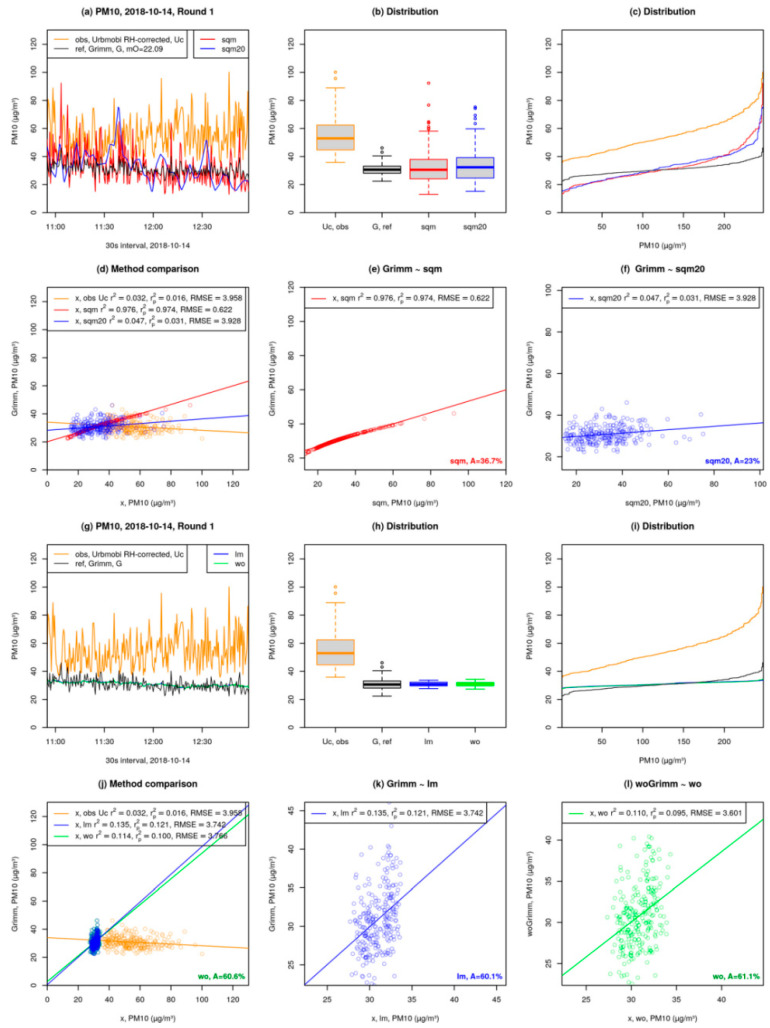
Factsheet for PM10 calibration of URBMOBI 3.0 with GRIMM 1.109 as the reference; an example of a measurement round at Berlin-Hermsdorf. The UBRMOBI 3.0 data obtained after calibration using the quantile mapping method has an accuracy of 36.7% in this example. Details for subfigures are provided in the text.

The following steps were performed for calibration (Figure 7):

1. The data sets were checked for outliers and inconsistencies due to manual or electrical errors. The first and the last 1% quantile of the URBMOBI 3.0 data are considered as outliers and removed.
2. Low performance of LCS due to RH is an issue repeatedly discussed in different studies on LCS. To compensate for the effect of aerosol hygroscopicity, the method described by Crilley et al. (2018) [50] wherein a correction factor “C”, derived based on the Köhler’s theory [51], is used. Crilley et al. (2018) [50] state that for a situation with RH < 60% a calibration against suitable reference instruments is sufficient. In the experiments we conducted, RH ranged from 50% to 85%. Therefore, it was decided to use the correction factor based on Köhler’s theory for the entire dataset. The value of ĸ is assumed to be 0.4 since the measurements were carried out in an urban area similar to that of the study conducted in Crilley et al. (2020) [54]. The URBMOBI 3.0 data is corrected for relative humidity using the following Equations (3) and (4).
  (3) $$C=1+\frac{\frac{ĸ}{\rho_{p}}}{-1+\frac{1}{a_{w}}},$$
  where, $a_{w}$ is the measured relative humidity over 100, *ĸ* is equal to 0.4, and density of particle ($\rho_{p}$) is set to 1.65 g/mL. The *C*-factor is then applied to the measurement data using:
  (4) $$PM_{corr}=\frac{PM_{raw}}{C},$$
3. The difference between the medians of the URBMOBI 3.0 (Uc) data set corrected for the influence of humidity and the Grimm 1.109 (G) data set is subtracted from the URBMOBI 3.0 to bring the measurements into the same range as the Grimm 1.109 data and then labeled with the subscript “s”.
4. Two models, linear regression (lm) and quantile mapping (qm) are tested for calibration. Each model uses two approaches. The first approach uses 100% of the Grimm 1.109 concurrent dataset to calibrate the URBMOBI 3.0 data (G~Uc). The second approach limits the derivation of calibration parameters to 20% of the common data, 10% at the beginning and 10% at the end, to check whether the statistical quantities found in this way can be used to reliably adjust the 80% original data during the mobile measurement without parallel reference.
5. As an additional step, outliers that might have been missed in step “1” are identified after step “4” as outliers in a boxplot. These values are removed and the Grimm 1.109 and URBMOBI 3.0 (RH-corrected) is correlated again (G~Wo).
6. The accuracy of the corrected URBMOBI 3.0 data (Uc) is checked. Accuracy (A) in this case is the percentage of data points that are within ±10% of the Grimm 1.109 data point at the same measurement time after calibration.

Figure 7 shows the analysis and comparison of quantile mapping (Figure 7a–f) and linear model (Figure 7g–l) on a bicycle-based mobile measurement carried out in Berlin-Hermsdorf. A description of each plot in the factsheet is given below:

Plots (a) to (f) in Figure 7 provide corrections based on quantile mapping:

- Time series in 30 s interval (preprocessed): Observation (obs), is the URBMOBI 3.0 (U) data (shown in dark orange). Reference (ref) is the Grimm data (shown in black). “mO” denotes the offset between the median of Grimm and URBMOBI 3.0. “sqm” is the corrected URBMOBI 3.0 time series after applying the median offset and before quantile mapping (shown in red). “sqm20” is the same as sqm but using only 20% of the Grimm device data (first and last 10%; this procedure reduces the time resolution; shown in blue) (Figure 7a).
- Distribution of Grimm, URBMOBI 3.0, and corrected URBMOBI 3.0 data shown in Figure 7a as boxplots (Figure 7b).
- Distribution of Grimm, URBMOBI 3.0, and corrected URBMOBI 3.0 data as seen in Figure 7a (Figure 7c).
- Comparison of methods used for URBMOBI 3.0 correction shown in Figure 7a, including a regression line based on simple linear regression. “obs” refers to the correlation between original URBMOBI 3.0 and Grimm data. “sqm” is the correlation between corrected URBMOBI 3.0 data using median offset before quantile mapping and the Grimm data (shown in dark orange). “sqm20” is same as sqm but with 20% of the Grimm data (first and last 10%; fewer data points) as reference. $r^{2}$ is the correlation coefficient of the correlation between Grimm and the corrected URBMOBI 3.0 data. $r_{p}^{2}$ denotes predicted $r^{2}$ based on the same data (Figure 7d).
- Correlation between sqm and Grimm (same as sqm in Figure 7d). “A” provides the accuracy of corrected URBMOBI 3.0 data. It is defined as the percentage of data points that are within the range of ±10% of the Grimm data point at the same measurement timestamp (Figure 7e).
- Correlation between sqm20 and Grimm (same as sqm in d) (Figure 7f).

The plots (g) to (l) in Figure 7 detail corrections based on multi-linear regression:

- Time series in preprocessed 30s interval: Observations (obs, URBMOBI 3.0) are shown in dark orange. Reference (ref, Grimm) is shown in black. “lm” refers to the corrected URBMOBI 3.0 data based on multi-linear regression (lm(G~U + RH + T)) wherein the intercepts and coefficients are gathered using a 5-min mean of URBMOBI 3.0 and Grimm data. Intercepts and coefficients are applied to the original URBMOBI 3.0 in the 30 s interval are shown in blue. For URBMOBI 3.0 data that was already RH-corrected with the C-factor RH was not considered for the multi-linear regression. “wo” is the same as lm, but with the outliers removed before calculating the intercepts and coefficients over a 5-min mean of both URMOBI 3.0 and Grimm data. Outliers are based on 30 s interval data: >1.5·Inter quartile range (IQR) as shown in light green (Figure 7g).
- Distribution of Grimm, URBMOBI 3.0, and corrected URBMOBI 3.0 data as boxplots as shown in Figure 7g (Figure 7h).
- Distribution of Grimm, URBMOBI 3.0, and corrected URBMOBI 3.0 data shown in Figure 7g (Figure 7i).
- Comparison of methods used for URBMOBI 3.0 correction shown in Figure 7g including a regression line based on simple linear regression: obs–correlation between URBMOBI 3.0 and Grimm data (shown in dark orange); lm–correlation between Grimm and corrected URBMOBI 3.0 based on 5-min means and Grimm data (shown in blue); wo–correlation between Grimm and corrected URBMOBI 3.0 data without outliers (shown in green). For a description of *r*^2^ and RMSE see Figure 7d with x as either obs, lm, or wo. $r^{2}$ and $r_{p}^{2}$ are not the correlation coefficients of the multi-linear model which was used for corrections). The accuracy (A) for correction without outliers was calculated using the same method as described for Figure 7e (Figure 7j).
- Same as Figure 7j, but zoomed in for lm. Accuracy of correction with outliers was calculated using the same method as described for Figure 7e (Figure 7k).
- Correlation between wo and Grimm-without-outliers (woGrimm). Accuracy of correction without outliers compared to Grimm without outliers was calculated using the same method as described for Figure 7e (Figure 7l).

**Figure 8:**
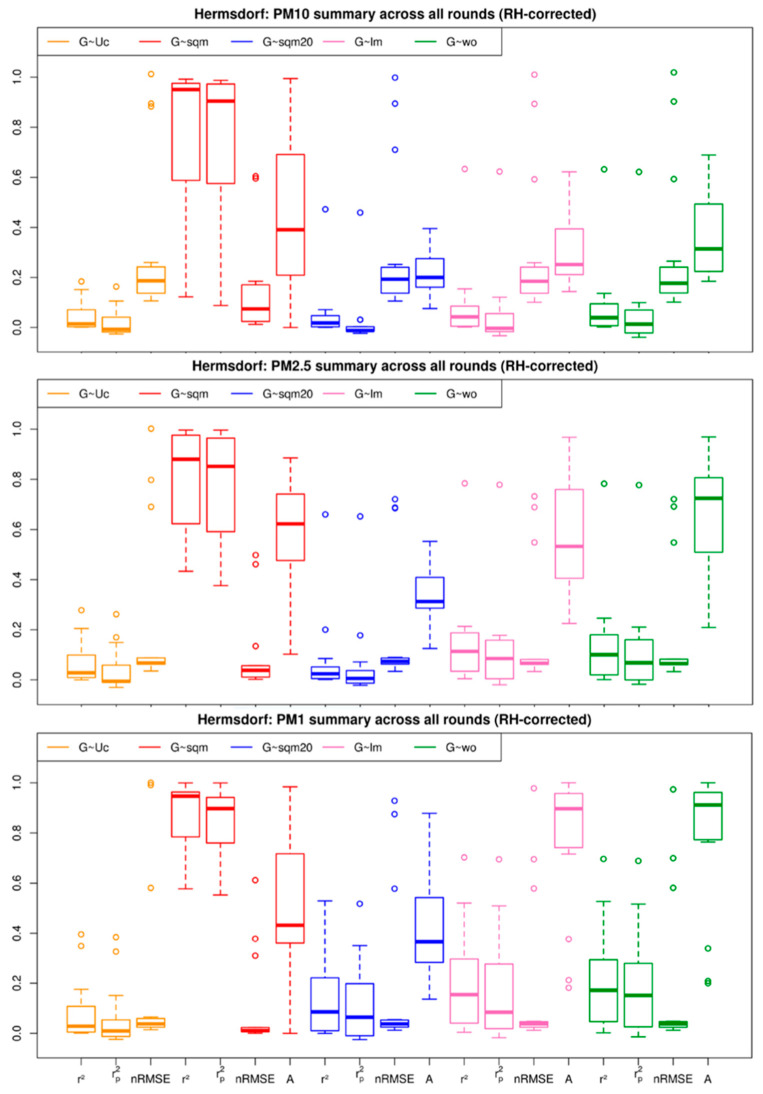
Summary of different correction methods, namely coefficients of determination (*r*²), the predicted coefficient of determination ($r_{p}^{2}$), and the normalized mean square of deviation (nRMSE) for RH corrected PM10, PM2.5 and PM1, for the measurement campaign in Berlin-Hermsdorf. The Accuracy (A) is also provided for the correlations between the Grimm 1.109 and URMBOBI 3.0 raw data (G~Uc), Grimm 1.109 and URBMOBI 3.0 corrected data using quantile method with 100% data (G~sqm), Grimm 1.109 vs. URBMOBI 3.0 corrected using quantile method with 20% data (G~sqm20), Grimm vs. URBMOBI 3.0 corrected with linear regression (G~lm), and Grimm 1.109 and URBMOBI 3.0 with all outliers removed (G~wo).

**Figure 9:**
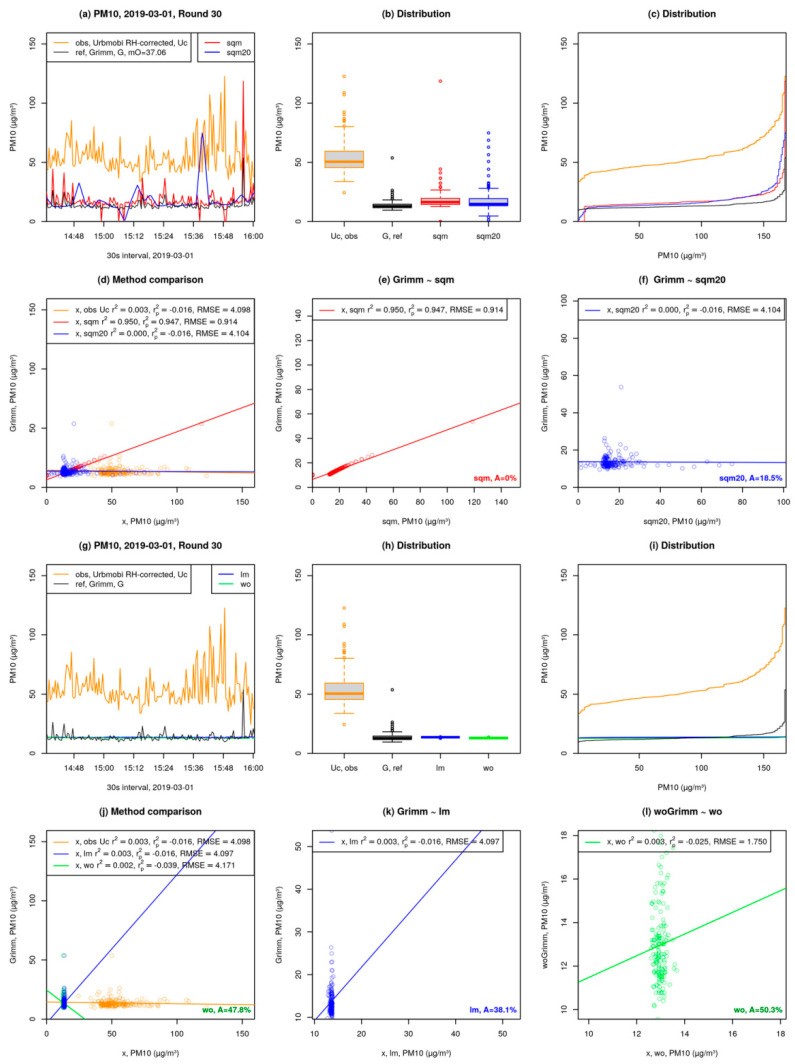
Factsheet for PM10 calibration of URBMOBI 3.0 with Grimm 1.109 as the reference; an example of a measurement round at Berlin-Hermsdorf with an accuracy of zero percent calculated after the calibration using the Quantile mapping method. Details for the subfigures are provided in the text under Section 3.3 and are the same as in Figure 7.

Although the linear regression model (lm) seems to look better (Figure 7a,g), it is also apparent in comparing the two figures that the spatial and temporal variability is lost in linear regression. Each measurement round is individually calibrated and a factsheet similar to Figure 7 is generated. Similar patterns are observed for most of the measurement rounds. Therefore, the quantile assignment is considered as the superior method. Figure 8 summarises all the rounds and generates a boxplot for each of the correction models considered for PM10, PM2.5 and PM1. It also shows the summary of the comparison between the different models and their respective coefficients of determination:

(5) $$r^{2}=1-\sum_{i=1}^{n}\frac{e_{i}^{2}}{\sum_{i=1}^{n}{\left(U_{i}-U\right)}^{2}},$$

where the predicted coefficient of determination ($r_{p}^{2}$), and the normalized mean square of deviation (nRMSE) were:

(6) $$nRMSE_{i}=\frac{RMSE_{i}}{RMSE_{max}}-RMSE_{min},$$

for all measurement rounds at the Hermsdorf site. The $r^{2}$ measures how well the model explains the given data and is dependent on the number of independent, explaining variables. When the number of independent variables and polynomial terms increases, it customizes itself to fit the peculiarities and random noise in the sample instead of reflecting the entire population. It also does not predict what would happen to the chosen model when it is used to calculate a different data set. The $r_{p}^{2}$ on the other hand, provides a good fit for the given data. Additionally, it can determine how well a regression model can make predictions [68,69]. This combined with nRMSE is used to assess the best model for calibrating LCS data on a mobile platform. In this respect, higher $r_{p}^{2}$ and/or a lower nRMSE indicate a better model. Figure 8 shows that the correction procedures work in similar ways for PM10, PM2.5 and PM1. Quantile mapping is significantly better than all other correction models for all three pollutants. The “sqm20” and lm methods produce better results for PM1 than for PM10 and PM2.5. It can also be concluded that it is important to not only assess mobile data with respect to its statistics (Figure 8) but also to check the time series (Figure 7 and Figure 9) in order to account for possible impacts of spatial characteristics.

It is important to note that each round in the Hermsdorf measurement campaign has been calibrated and corrected individually. This in itself speaks for the inherent difficulties in calibrating any data acquired with a LCS on a mobile platform. Although the qm method seems to be a good option for calibrating LCS on a mobile platform, it does not work similarly for all the measurement rounds. This can be seen in Figure 9, wherein the URBMOBI 3.0 data is corrected to within the range of the Grimm data but the accuracy is 0% (Figure 9e).

## 4. Summary and Conclusions

Despite their shortcomings, LCS have become an alternative for those who cannot afford expensive devices or/and for those who want to expand AQMS networks. The field of LCS is one with many open questions, especially concerning the behaviour of sensors under changes in temperature and relative humidity, and aging. To increase the environmental awareness of people or to provide qualitative analysis of the AQ at a high spatial resolution, the data provided by most of the LCS are sufficient. However, quantitative measurements using OEMs are not suitable without at least additional temperature and humidity measurements and bias corrections as well as cross-calibration with existing reference stations using the conventional established equipment of AQMS networks.

It appears that laboratory or co-location experiments alone might be insufficient to determine the accuracy of measured values and the reliability and characteristics of the sensors. Therefore, calibration of the sensors on a mobile platform has to be performed using at least partly parallel measurements with quality assured standard devices. At this stage, it is not recommended to only use LCS in mobile setups, especially for quantitative measurements. However, if and when used, stationary calibrations are insufficient due to rapid changes in environmental conditions such as temperature and humidity. However, using quantile mapping wherein 20% of the data is used to correct the rest of the data set can come in handy to avoid relying on a reference-grade device for the entire measurement time. Setting up two or more reference stations or using existing AQMS stations along the measurement route and measuring next to them for a couple of minutes, and using this as a “20%” data to calibrate the LCS, could provide the same effect.

In addition, the effect of wind speed and the inaccuracies resulting from vibrations need to be further investigated. Moreover, as the expert interviews have revealed, age-related drift of the sensors and their service life must be accounted for.

Datasheets provided by the manufacturers are quite often insufficient to assess the characteristics of the sensors. A variety of studies from independent researchers, scientists, and public authorities have developed testing and calibration methods to compensate for that. However, the lack of uniformity in the testing of sensors partly invalidates the results or makes it hard to work out comparisons between studies and across different sensors. The number of calibrations to be carried out during a measurement campaign is also not easy to assess. This calls for establishing a seal of approval based on standardised testing procedures, and the development and establishment of general guidelines for the use of such sensors in AQ networks both for general monitoring and scientific measurement campaigns. Further, a common platform for users of LCS for communication, information, and idea exchange is indispensable as the circle of users is constantly growing in private, commercial, and scientific applications.

## Figures and Tables

**Table 1:** List of low-cost PM sensors with the name of the manufacturer, model number, dimensions in mm, measurement principle used, the measurement and detection ranges, time resolution (T.R.), and the approximate cost.

Manufacturer	Model	Dimension	Principle	Measurement and Detection Range	T.R.	Cost
Alphasense Ltd. (Great Britain)	OPC-N2	75 × 63.5 × 60	L.S.S.	0.38–17 µm, 16 Channels (Number concentration), PM1, PM2.5, PM10	1.4 s	N.A.
	OPC-N3	75 × 63.5 × 60	L.S.S.	0–2000 µg/m^3^ 0.35–40 µm, 24 Channels (Number concentration), PM1, PM2.5, PM10, Temperature and RH	1 s	415 €
	OPC-R1	72 × 25.5 × 21.5	L.S.S.	0.35–12.4 µm 16 Channels (Number concentration), PM1, PM2.5, PM10, Temperature and RH	1 s	210 €
Dylos Corp (USA)	DC1700 PM PM2.5/PM10 AQM	17.8 × 11.4 × 7.6	L.S.S.	0–10^6^ Particle/cm^3^ >0.5 and >2.5 µm and PM2.5 and PM10 in µg/m^3^	60 s	420 €
Honeywell (USA)	HPMA115SO-XXX	36 × 43 × 24	L.S.S.	0–1000 µg/m^3^ PM2.5 in µg/m^3^ (PM10 in µg/m^3^ with additional programming)	N.A.	30 €
Met One (USA)	831 Aerosol Mass Monitor	159 × 92.2 × 50.8	Photometer	0–1.000 µg/m^3^ >0.1 µm	60 s	1700 €
Nova Fitness (China)	SDS011	71 × 70 × 23	L.S.S.	0–999.9 µg/m^3^ 0.3–10 µm	1 s	30 €
	SDS018	59 × 45 × 20	L.S.S.	0–999.9 µg/m^3^ 0.3–10 µm	1 s	30 €
	SDS198	71 × 70 × 23	L.S.S.	0–20 mg/m^3^ 1–100 µm	1 s	30 €
Plantower (China)	PMS 1003	65 × 42 × 23	L.S.S.	0–500 µg/m^3^ 0.3–1.0; 1.0–2.5; 2.5–10 µm in three channels	N.A.	15 €
	PMSA003	38 × 35 × 12	L.S.S.	0–500 µg/m^3^ 0.3–1.0; 1.0–2.5; 2.5–10 µm in three channels	N.A.	20 €
	PMS 3003	65 × 42 × 23	L.S.S.	0.3–1.0; 1.0–2.5; 2.5–10 µm in three channels	N.A.	20 €
	PMS 5003	N.A.	L.S.S.	N.A.	N.A.	15 €
	PMS 7003	N.A.	L.S.S.	N.A.	N.A.	20 €
Samyoung (South Korea)	PSML(LPO)	N.A.	Photometer	0–900 μg/m^3^ PM2.5 and PM1	1 s	N.A.
	DSM501A	N.A.	Photometer	>1 µm	N.A.	17 €
Sensiron (Switzerland)	SPS30	40.6 × 40.6 × 12.2	L.S.S	1–1000 μg/m^3^ PM1, PM2.5, PM4, and PM10 (Mass) PM0.5, PM1, PM2.5, PM4 and PM10 (Particle number)	N.A.	40 €
Sharp (Japan)	GP2Y1010AU0F	46 × 30 × 17.6	Photometer	N.A.	N.A.	12 €
	DN7C3CA006	51 × 53 × 40	Photometer	25–500 µg/m^3^	N.A.	22 €
Shinyei (China)	PPD42NJ	59 × 45 × 22	Photometer	>1 µm	N.A.	25 €
	PPD60PV-T2	88 × 60 × 20	Photometer	>0.5 µm	N.A.	N.A.
	PPD20V	88 × 60 × 20	Photometer	>1 µm	N.A.	N.A.
	PPD71	34 × 30 × 28	Photometer	>0.5 µm	N.A.	N.A.
Winsen (China)	ZH03B	50 × 32.4 × 21	Photometer	0–1000 µg/m^3^	N.A.	32 €

**Table 2:** Electrical and performance characteristics of low-cost PM sensors with the name of the manufacturer, model number, operating temperature (T) and relative humidity (RH) range, nominal voltage and maximum power consumption, uncertainty, sensor life, availability of calibration, and the reaction time in seconds (s); “n.c.” stands for “non condensing”.

Manufacturer	Model	T and RH	Voltage and Power Consumption	Uncertainty	Sensor Life	Integrated Calibration	Reaction Time
Alphasense Ltd. (Great Britain)	OPC-N2	−10–50 °C 0–95% (n.c.)	4.8–5.2 V 0.90 W	N.A.	N.A.	No	N.A.
	OPC-N3	−10–50 °C 0–95% (n.c.)	4.8–5.2 V 0.90 W	N.A.	N.A.	Yes	N.A.
	OPC-R1	−10–50 °C 0–95% (n.c.)	4.8–5.2 V 0.48 W	N.A.	N.A.	Yes	N.A.
Dylos Corp (USA)	DC1700 PM PM2.5/PM10 AQM	N.A.	110 V or Battery	N.A.	N.A.	Yes	6 s
Honeywell (USA)	HPMA115SO-XXX	−10–50 °C 0–95% (n.c.)	5 ± 0.2 V 0.40 W	±15 µg/m^3^ (0–100 µg/m^3^) ±15% (100–1000 µg/m^3^) at 25 ± 5 °C	20,000 h	No	<6 s
Met One (USA)	831 Aerosol Mass Monitor	0–50 °C	100–240 V to 8.4 V(AC/DC) Li-Battery, rechargeable	N.A.	N.A.	Yes	N.A.
Nova Fitness (China)	SDS011	−20–60 °C <70%	5 ± 0.2 V 0.40 W	Max. ±15% and ±10 µg/m^3^ at 25 °C, 50% RH	N.A.	No	<10 s
	SDS018	−20–60 °C <70%	5 ± 0.2 V 0.35 W	Max. ±15% and ±10 µg/m^3^ at 25 °C, 50% RH	N.A.	No	<10 s
	SDS198	−20–60 °C <70%	5 ± 0.2 V 0.40 W	Max. ±20% and ±30 µg/m^3^ at 25 °C, 50% RH	N.A.	No	<10 s
Plantower (China)	PMS 1003	N.A.	5 ± 0.2 V	N.A.	N.A.	No	N.A.
	PMSA003	−10–60 °C 0–99%	5 ± 0.5 V	±10 µg/m^3^ (0–100 µg/m^3^) ±10% (100–500 µg/m^3^)	N.A.	No	10 s
	PMS 3003	N.A.	5 ± 0.2 V	N.A.	N.A.	No	N.A.
	PMS 5003	N.A.	N.A.	N.A.	N.A.	No	N.A.
	PMS 7003	N.A.	N.A.	N.A.	N.A.	No	N.A.
Samyoung (South Korea)	PSML(LPO)	−10–65 °C <95% (n.c.)	5.0 ± 10% V 0.43 W	N.A.	N.A.	No	N.A.
	DSM501A	−10–65 °C	5 ± 0.5 V	N.A.	N.A.	No	N.A.
Sensiron (Switzerland)	SPS30	−10–60 °C	5 ± 0.5 V 0.30 W	±10 μg/m^3^ (0 to 100 μg/m^3^) ±10% (100–1000 μg/m^3^)	>8 Years	Yes	N.A.
Sharp (Japan)	GP2Y1010AU0F	−10–60 °C 10–90%	5 ± 0.5 V 0.10 W	N.A.	N.A.	No	N.A.
	DN7C3CA006	−10–60 °C 10–90%	5 ± 0.25 V 0.10 W	N.A.	N.A.	No	N.A.
Shinyei (China)	PPD42NJ	0–45 °C <95% (n.c.)	5 ± 0.2 V	N.A.	N.A.	No	N.A.
	PPD60PV-T2	0–45 °C <95% (n.c.)	5 ± 0.2 V	N.A.	N.A.	No	N.A.
	PPD20V	0–40 °C <95% (n.c.)	5 ± 0.2 V	N.A.	N.A.	No	N.A.
	PPD71	−10–60 °C <95% (n.c.)	5 ± 10% V	N.A.	N.A.	No	N.A.
Winsen (China)	ZH03B	−10–50 °C 0–85% (n.c.)	5 ± 0.1 V 0.60 W	N.A.	3 Years	No	N.A.

## Data Availability

The data presented in this study are available on request from the corresponding author.

## Appendix A

**Table A1:** Experts contacted as a part of a project on developing a communication strategy for citizen science projects and the general public on the usage of LCS for the German Environmental Agency (Umweltbundesamt, UBA).

Name	Organisation	City, Country	Project	Sensor
Saúl García	Instituto de Salud Carlos III	Madrid, Spain	ICARUS	Uhoo, ioTech-portable PM sensor
David Kocman	Department of Environmental Sciences, Institut “Jozes Stefan”	Ljubljana, Slovenia	ICARUS	Uhoo, ioTech-portable PM sensor
Ondřej Mikeš	RECETOX, Masaryk University	Brno, Czech Republic	ICARUS	Uhoo, ioTech-portable PM sensor
Bernd Laquai	University of Stuttgart	Stuttgart, Germany	Feasibility study, for the use of sensors in the context of an epidemiological study.	Sensors from Alphasense Ltd., SDS011
Andreas Madsasck	Ok Lab	Stuttgart, Germany	SENSOR.COMMUNITY	SDS011
Núria Castell	Norwegian Institute for Air Research (NILU)	Oslo, Norway	iFLINK European Citizen Science Association Working Group	Alphasense Ltd., SDS011
Robert Heinecke	Breeze Technology	Hamburg, Germany	The state of Hennef, The state of Moers, and own Projects	Purchased from different companies
Hester Volten	National Institute for Public Health and the Environment, Ministry of Health, Welfare and Sport.	Netherlands	Multiple Citizen Science Projects	SDS011, Alphasense Ltd.
Helge Simon & Lorenz Harr	Johannes Gutenberg–Universität Mainz	Mainz, Germany	Own Project	Alphasense Ltd.
Michel Gerboles & Annette Borowiak	European Commission Joint Research Centre	Ispra, Italy	AirSensEUR, AQUILA	Different sensor boxes
Christof Asbach	Institut für Energie and Umwelttechnik (IUTA) e.V.	Duisburg, Germany	Own Project	SDS011, sensors from Alphasense Ltd.
Dieter Klemp & Robert Wagner	Forschungszentrums Jülich	Juelich, Germany	Sensoren zur Messung von Aerosolen and reaktiven Gasen and Analyse ihrer Auswirkung auf die Gesandheit (SMARAGD)	SDS011, Alphasense Ltd. (NO2-B43F, NO-B4, OX-B431, CO-B4)
Anke Lükewille	European Environmental Agency (EEA)	Copenhagen, Denmark	EEA report on Assessing air quality through citizen science	
Panagiota Syropoulou	DRAXIS, hackAIR	Greece	hackAIR	Air quality awareness project that eventually leads to the use of SDS011.
Erika von Schneidemesser	Institute for Advanced Sustainability Studies (IASS)	Potsdam, Germany	Urban Climate Under Change [UC]²	Earth Sense (Zephyr)
Andreas Phillip	Universität Augsburg	Augsburg, Germany	Smart AQnet	SDS011, Alphasense Ltd.
Alberto Gotti	EUCENTRE, Department of Risk Scenarios	Pavia, Italy	ICARUS	Uhoo, ioTech-portable PM sensor
Danielle Vienneau	Swiss TPH	Basel, Switzerland	ICARUS	Uhoo, ioTech-portable PM sensor
Markus Pesch	Grimm Aerosol Technik GmbH	Muldestausee, Germany	Smart AQnet	Alphasense OPC N3
Stefan Hogekamp	PALAS	Karlsruhe, Germany	Own Project	Own technology
M. M. Prada	Bosch	Renningen, Germany	Own Project	Different sensors
